# Genomic Analysis of Porcine Reproductive and Respiratory Syndrome Virus 1 Revealed Extensive Recombination and Potential Introduction Events in China

**DOI:** 10.3390/vetsci9090450

**Published:** 2022-08-23

**Authors:** Fang Yu, Liqiang Liu, Xiaoxiao Tian, Ligong Chen, Xinyi Huang, Yue Sun, Yi Yan, Zhijun Tian, Xuehui Cai, Di Liu, Tongqing An

**Affiliations:** 1 State Key Laboratory of Veterinary Biotechnology, Harbin Veterinary Research Institute, Chinese Academy of Agricultural Sciences, Harbin 150008, China; 2 College of Life Sciences and Food Engineering, Hebei University of Engineering, Handan 056006, China; 3 College of Veterinary Medicine, Hebei Agricultural University, Baoding 071051, China; 4 Computational Virology Group, Center for Bacteria and Viruses Resources and Bioinformation, Wuhan Institute of Virology, Chinese Academy of Sciences, Wuhan 430071, China; 5 CAS Key Laboratory of Special Pathogens and Biosafety, Wuhan Institute of Virology, Chinese Academy of Sciences, Wuhan 430071, China

**Keywords:** porcine diseases, PRRSV-1, genetic diversity, phylogenetic analysis, recombination, RT-qPCR

## Abstract

**Simple Summary:**

Porcine reproductive and respiratory syndrome, caused by the porcine reproductive and respiratory syndrome virus, is considered one of the most devastating swine diseases worldwide. Porcine reproductive and respiratory syndrome virus 1 was first isolated in China in 2006, and there have been few reports concerning its genetic characteristics in China. We hope to find out the regularity of genetic diversity, recombination, and evolution of the virus by analyzing all available genomic sequences during 1991–2018. We found that high-frequency recombination regions were concentrated in non-structural protein 2 and structural proteins 2 to 4 and extensive deletions in non-structural protein 2; phylogenetic analysis revealed four independent introductions in China. Our results suggest that attention should be paid to the prevention and control of porcine reproductive and respiratory syndrome virus 1 and the rational use of vaccine strains. These results will help us to understand the recombination of porcine reproductive and respiratory syndrome virus and strengthen viral inspection before mixing herds of swine to reduce the probability of novel recombinant variants. Moreover, our study might form the basis of monitoring and control measures to prevent the spread of this economically important virus.

**Abstract:**

Porcine reproductive and respiratory syndrome (PRRS), caused by the PRRS virus (PRRSV), is considered one of the most devastating swine diseases worldwide. PRRSV-1 was first isolated in China in 2006. However, there were few reports concerning the genetic characteristics of PRRSV-1 in China. In this study, three PRRSV-1 strains (HL85, HeB3, and HeB47) were detected by a general RT-qPCR method from clinical samples in 2018. HeB47 was identified as a recombinant between the BJEU06-1 and CReSA228-like strains. To further analyze the recombination and deletion features of PRRSV-1, all the available 88 complete genome sequences (isolated in 19 countries) from 1991 to 2018 in GenBank were analyzed. The high-frequency recombination regions were concentrated in NSP2 and GP2 to GP4. More importantly, phylogenetic analysis of PRRSV-1 revealed four independent introductions in China. Therefore, it is necessary to strengthen the important monitoring of breeding pigs and pork products and epidemiological surveys on pig farms to prevent the further spread of PRRSV-1.

## 1. Introduction

Porcine reproductive and respiratory syndrome (PRRS), caused by the PRRS virus (PRRSV), is considered one of the most economically challenging diseases for pig industries worldwide [1,2]. The disease, characterized by reproductive failure in sows and respiratory disorders and growth retardation in growing pigs, was first described in the United States in 1987 [3]. PRRSV is an enveloped single-stranded positive-sense RNA virus belonging to the family *Arteriviridae* within the order *Nidovirales* [4]. The PRRSV genome is approximately 15 kb in length and contains at least 10 open reading frames (ORFs), including ORF1a, ORF1b, ORF2a, ORF2b, ORF3 to 7, and ORF5a [5]. ORF1a and ORF1b encode two polyproteins (pp1a and pp1ab) [6], whereas ORFs 2 to 7 encode eight viral structural proteins (GP2a, E, GP3, GP4, ORF5a, GP5, M, and N) [5,7,8]. GP2, GP3, and GP4 are incorporated as multimeric complexes in the envelopes of PRRSV [9,10]. 

PRRSV has been classified into PRRSV-1 and PRRSV-2 [11,12]. According to a recently proposed new taxonomic scheme, those have been classified as two species within the genus Betaarterivirus: *Betaarterivirus suid 1* and *Betaarterivirus suid 2*, respectively. Based on phylogenetic methods, PRRSV-1 can be divided into four subtypes: subtypes 1, 2, 3, and 4 [13]. Non-structural protein 2 (NSP2) is the most variable non-structural protein, with only 32% identity shared between subtypes [14]. GP3 shares the lowest degree of identity with PRRSV-1 and is recognized as one of the most heterogeneous proteins [15]. GP5 is the major glycosylated protein and has a high variability within PRRSV. Recombination events were detected in PRRSVs, which can be found between two wild-type PRRSVs [16], between wild-type PRRSVs and modified live vaccine (MLV) strains [17,18], and between the two MLV strains VP-046BIS and DV [19]. In the present study, we sequenced three PRRSV-1 strains and analyzed them with 88 complete genome PRRSV sequences. Complex NSP2 deletion polymorphisms and extensive recombination were explored in the present study. The results suggest that attention should be paid to the prevention and control of PRRSV-1 and the rational use of vaccine strains.

## 2. Materials and Methods

### 2.1. Sample Processing and Sequencing 

A total of 260 clinical samples, including lungs, livers, lymph nodes, and serum, were collected from different pig farms experiencing respiratory disorders, emaciation, growth retardation, and asthma in pigs in nine provinces (Heilongjiang, Jilin, Inner Mongolia, Hebei, Henan, Shanxi, Sichuan, Chongqing, and Jiangsu) in China from 2017 to 2018. Tissues from pigs were homogenized for RNA extraction by using the QIAamp Viral RNA Mini Kit (QIAGEN, Hilden, Germany), in accordance with the manufacturer’s instructions. Reverse transcription-PCR (RT-PCR) was performed according to the previously described method [20]. 

The complete genomes of three positive samples by RT-PCR were sequenced. Specifically, the samples HeB3 and HeB47 from Hebei were amplified with 16 overlapping fragments by RT-PCR [20]. The amplified PCR products were gel-purified using a Gel Extraction Kit (OMEGA, Norcross, GA, USA), cloned into the pMD18-T vector (TaKaRa, Dalian, China), and then at least five independent clones were sequenced using the Sanger method (Invitrogen, Beijing, China). Genome assembly and sequence alignments were performed using SeqMan and MegAlign in Lasergene (Version 7.0, DNASTAR Inc., Madison, WI, USA), respectively. The other sample, HL85, collected from Heilongjiang, was subjected to next-generation sequencing (NGS) on a HiSeq platform (Illumina, San Diego, CA, USA), as previously described [21]. 

### 2.2. Virus Isolation

Three positive samples were homogenized in Dulbecco’s modified Eagle’s medium (DMEM, Gibco) and the suspensions were passed through 0.22 μm filters to inoculate porcine alveolar macrophages (PAMs) and Marc-145 cells. The supernatants were harvested at 72 h post-infection (hpi), designated as passage 1 (P1), and stored at −80 °C. Subsequently, P1 viruses were serially passaged two times (P2-P3). The culture suspensions of P3 from PAM and Marc-145 cells were used to detect PRRSV-1 by RT-PCR. 

### 2.3. Dataset

All available full-length genomes of PRRSV-1 (*n* = 88) during 1991–2018 (Table S1) and 4 reference PRRSV-2 full-length genomes were downloaded from GenBank to construct a phylogenetic tree based on full-length genomes. Ninety-one ORF5 split from full-length sequences, all available Chinese ORF5 partial genomes during 1991–2018 (*n* = 31) shown in Table S2 and 34 reference ORF5 genomes (PRRSV-1 = 28; PRRSV-2 = 6) from GenBank to construct ORF5-based phylogenetic tree. To study NSP2 deletion polymorphisms, NSP2 amino acid sequences were derived from 91 full-length genomic sequences, which represented the diversity of the viruses from 1991 to 2018. 

### 2.4. Recombination Analysis

Recombination analysis was performed using RDP v.4.96 with default parameters [22], and recombination events were indicated when three out of seven methods (RDP, BOOTSCAN, MAXCHI, CHIMAERA, 3SEQ, GENECONV, and SISCAN) reported recombination signals. Only the recombination events with sufficient evidence (excluding partial or trace evidence in the results) and both parental strains clearly identified were kept. The detected recombination events were further analyzed using SimPlot v3.5 (Ray, S. C, Baltimore, MD, USA) [23], with a window size of 500 bp and step size of 20 bp. The final recombination event was determined with the support of both the RDP and SimPlot software. The recombination parental strains and breakpoint locations were determined based on the RDP and SimPlot results. The recombination frequency for each site was the percentage of recombination events that occurred at a specific site in proportion to the total number of recombinants. 

### 2.5. NSP2 Polymorphic Patterns

To determine the various insertion and deletion (indel) patterns of NSP2, an optimized sequence alignment was obtained according to the following procedures. First, 91 complete genome sequences of all PRRSV-1 from 1991 to 2018 were aligned using MAFFT 7 [24]. Second, the alignment was split to obtain NSP2 sequences based on the Lelystad virus (LV) annotation. Third, the re-alignment of NSP2 was based on the amino acid sequences created by Clustal W in Lasergene (Version 7.0, DNASTAR Inc., Madison, WI, USA). All deletions and insertions were labeled, and the percentage of deletions at each site was calculated (the percentage of amino acid deletions at each site relative to the total number of strains). 

### 2.6. Phylogenetic Analysis

Multiple sequence alignments were generated using MAFFT 7. Phylogenetic trees were constructed in RAxML-NG using the GTRGAMMA nucleotide substitution model with 1000 bootstrap replicates [25]. According to the classification criteria for PRRSV-1 in previous studies [13,26], ORF5 sequences were classified into different subtypes, corresponding to subtypes 1, 2, 3, and 4.

### 2.7. Quantitative Reverse-Transcription PCR (RT-qPCR)

#### 2.7.1. Design of Primers and Probe

All available genome sequences (*n* = 91) of PRRSV-1 were obtained from the GenBank database and clinical samples, and aligned using DNASTAR (DNASTAR Inc., Madison, WI, USA) to identify the conserved region of PRRSV-1. Specific primers and probe for PRRSV-1 were designed according to the conserved region of the ORF6 gene as follows: sense primer, 5′-CTGTGAGAAAGCCCGGACT-3′; antisense primer, 5′-GGCCATACTTGACGAGGTTA-3′; probe, 5′-FAM-TGGGCGGCAAYCGAGCTGT-MGB-3′. Primers and probe were synthesized by Comate Bioscience Co., Ltd. (Jilin, China).

#### 2.7.2. RT-qPCR Assay

The RT-qPCR assay was conducted using the One Step PrimeScript™ RT-PCR Kit (TaKaRa), with some modifications. After optimization, the 20-μL reaction mixtures contained 2 μL viral RNA or DNA, 10 μL 2X One-Step RT-PCR Buffer Ⅲ, 0.4 μL TaKaRa Ex Taq HS (5 U/μL), 0.4 μL PrimeScript RT Enzyme Mix Ⅱ, 0.4 μL ROX Reference Dye, 0.8 μL each pair of primers, 0.8 μL probe (10 μM) and 4.4 μL nuclease-free water. Thermal cycling conditions involved an initial 5 min incubation at 42 °C, then 95 °C for 10 s, followed by 40 cycles of 95 °C for 5 s and 57 °C for 20 s. The reaction was carried out using QuantStudio 5 Real-Time PCR System (Real-Time PCR System, Waltham, MA, USA).

#### 2.7.3. Generation of the Standard Curve

To prepare a positive plasmid to serve as the standard sample, the majority sequence of the ORF6 gene based on the alignments of PRRSV-1 was synthesized and cloned into a pUC57 vector (Comate Bioscience Co., Ltd., Jilin, China). The positive plasmid was verified by M13 sequencing primers to successfully introduce the ORF6 gene. The plasmid, pUC57-PRRSV-1-ORF6, was extracted by the Plasmid Mini Kit I (Omega, Norcross, GA, USA) and quantified by measuring OD260. The copy number was calculated according to the following formula: copy number = (ng × 6.02 × 10^23^ × 10^−9^)/[fragment length (bp) × 660]. Through ten-fold gradient dilution, the plasmid concentrations ranging from 2.5 × 10^1^ to 2.5 × 10^8^ copies/μL were required. 

#### 2.7.4. Sensitivity Analysis, Specificity Analysis, Reproducibility, and Repeatability Analysis

To identify the detection limit, the standard curves were generated using standard plasmids (2.5 × 10^1^–2.5 × 10^8^ copies/μL). To assess the specificity of RT-qPCR assay in the diagnosis of PRRSV-1, PRRSV-2 (including highly pathogenic-PRRSV, classical-PRRSV, and NADC30-like-PRRSV), porcine epidemic diarrhea virus (PEDV), porcine circovirus 2 (PCV2), classical swine fever virus (CSFV), and pseudorabies virus (PRV) were detected. For the reproducibility analysis, high, medium, and a low dose of standard plasmids (2.5 × 10^6^, 2.5 × 10^4^, and 2.5 × 10^2^ copies/μL) were tested by the RT-qPCR assay through three independent runs. For the repeatability analysis, the same serial dilutions of standard plasmids were tested by the RT-qPCR assay in triplicate within one run. The coefficient of variation (CV) was calculated to evaluate the results.

### 2.8. Nucleotide Sequence Accession Numbers

The three nucleotide sequences of PRRSVs sequenced in the present study have been deposited in GenBank (accession no. MN927227 to MN927229).

## 3. Results

### 3.1. Identification of PRRSV-1 Samples in Mainland China

Although there have been many reports about the prevalence of PRRSV-1 in China in recent years, the clinical detection rate is still low [20,27,28]. To provide insight into PRRSV-1 in China from 2017 to 2018, 260 clinical samples were collected from pig farms in nine provinces (Heilongjiang, Jilin, Inner Mongolia, Hebei, Henan, Shanxi, Sichuan, Chongqing, and Jiangsu). Outbreaks of PRRSV-1 have been reported in Heilongjiang and Inner Mongolia [18,20,27]. As the PRRSV-1 vaccine has not been approved in China and the clinical pathogenicity of the PRRSV-1 is not high, the pigs in these farms were not vaccinated. Among the 260 samples, there were three positive samples by RT-PCR, including two samples from pig farms in Hebei province, which had low growth uniformity, emaciation, and asthma in pigs. Another sample came from a pig farm in Heilongjiang province. The pigs were emaciated, shaggy, anorexic, and prone to lying down and slow growing. 

Viral isolation was performed twice in pulmonary alveolar macrophage (PAM) and Marc-145 cells, but no PRRSV was successfully isolated. Briefly, after PAM and Marc-145 cells passage for three times, cytopathic effect (CPE) and RT-PCR showed no proliferation of the virus. 

### 3.2. Genomic Sequence Analysis of Three PRRSV-1 Samples

To study the diversity of PRRSV-1, three clinical samples were sequenced using the NGS or Sanger method. Referring to previous sequencing and analysis methods [29], NGS sequencing results of the HL85 sample showed that the reads per million mapped reads (RPM) of PRRSV, Mycoplasma hyorhinis, Haemophilus parasui, and Mycoplasma hyopneumoniae were 302, 70, 41, and 11, respectively. The genome sizes of the samples HL85, HeB3, and HeB47 were 15,036, 15,084, and 15,113 nucleotides (excluding polyA), respectively. The complete genome, pp1a, pp1ab, GP2, GP3, GP4, GP5, M, and N proteins shared 88.4~93.8%, 78.7~92.3%, 96.6~98.4%, 90.4~94.8%, 78.9~92.9%, 89.5~91.2%, 87.6~91.6%, 96.5~98.3%, and 91.5~96.1% nucleotide and amino acid identity with the LV strain, respectively (Table 1). 

### 3.3. Deletion and Mutations of the Three PRRSV-1 Samples

**Figure 1:**
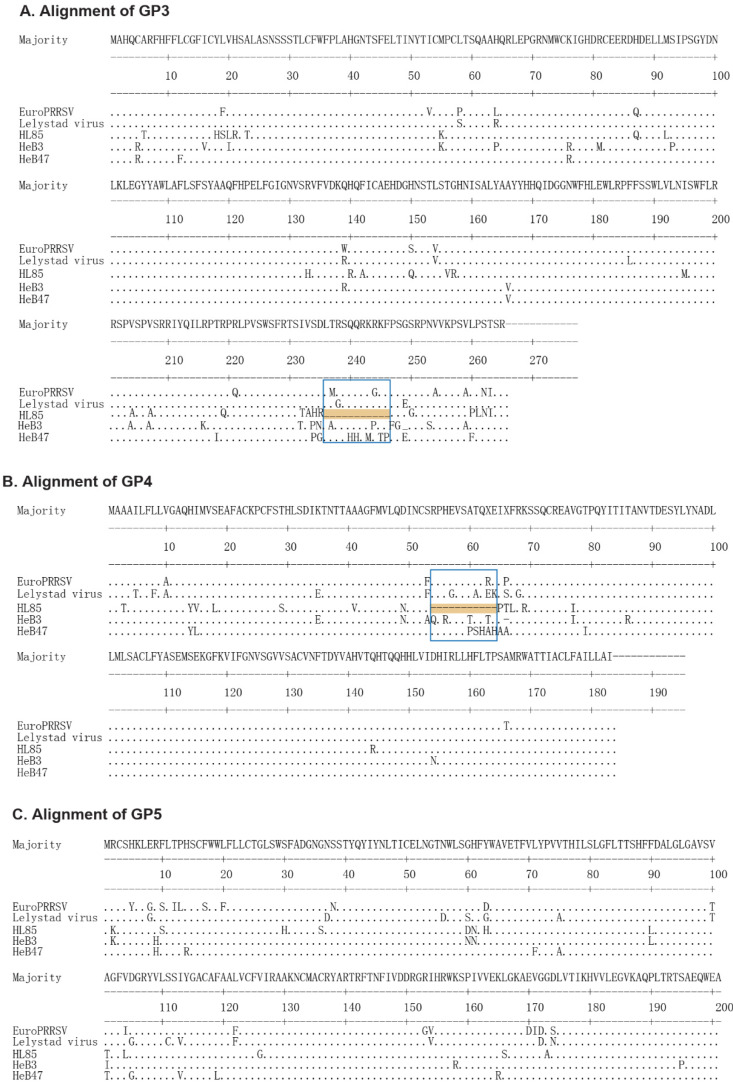
The amino acid sequences alignment of GP3, GP4, and GP5 structural proteins: (**A**) Alignment of the GP3 region of PRRSV-1 strains. The 11-aa continuous deletion is highlighted by the blue box. (**B**) Alignment of the GP4 region of PRRSV-1 strains. The 11-aa continuous deletion is highlighted by the blue box. (**C**) Alignment of the GP5 region of PRRSV-1 strains.

Amino acids (aa) in each protein region were compared with the representative EuroPRRSV (the first isolate of PRRSV-1 in North America) and LV (the PRRSV-1 prototype, the first isolate of PRRSV-1 in Europe). HL85 had 33 nucleotide (nt) deletions in the overlapping region of ORF3 and ORF4, which led to an 11-aa continuous deletion at aa position 236 to 246 and 54 to 64 of GP3 and GP4, respectively (Figure 1). Deletions in the overlapping region were commonly found in PRRSV-1 isolates, such as HKEU16, BJEU06-1, and NMEU09-1 isolates. There were a few irregular mutations in the amino acids of the GP5 region. The GP2, M, and N regions only have sporadic amino acid mutations (Figure S1).

### 3.4. Recombination Analysis of PRRSV-1 Samples 

**Figure 2:**
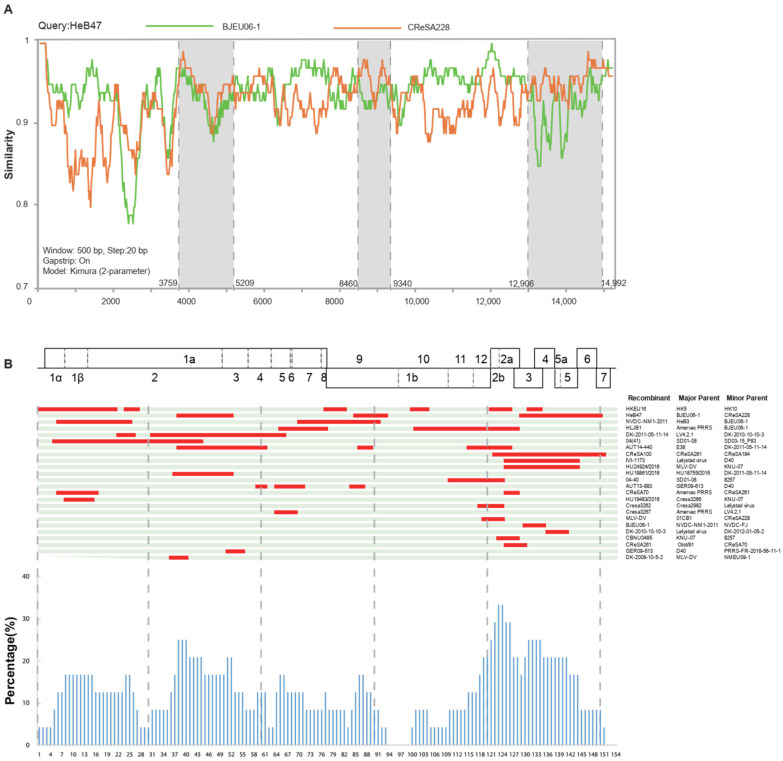
Map of the HeB47 recombinant and recombination patterns of genomes: (**A**) Genome scale similarity comparisons of HeB47 (query) with BJEU06-1 (green) and CReSA228 (orange). Recombination breakpoints are highlighted by the black dotted lines, with the locations indicated at the bottom. The background color of the major parental regions is white, whereas that of the minor parental regions is gray. (**B**) Map of the parental lineages of PRRSV-1 genomes. The top of the figure is the whole genome structure, with reference to the strain with the longest genome length of the 91 strains, in which the positions and boundaries of the major ORFs and NSPs within ORF1a and ORF1b are shown. Gray vertical dashed lines spaced 3000 bp apart were used to locate the recombination breakpoint and x-axis positions of the histogram. The name of each strain and parents are displayed on the right, with the corresponding major and minor parents shown in green and red on the map, respectively. The *x*-axis represents the PRRSV genome position and the *y*-axis represents the number of recombinations that occurred at a specific site in proportion to the total number of recombinants in a sliding window (per 100 nucleotide bases) centered on that position on the *x*-axis.

To test the possible recombination events of the three PRRSVs sequenced in the clinical samples, a similarity comparison with SimPlot was performed. The analysis revealed that HeB47 was a recombinant of the BJEU06-1 strain and CReSA228-like strain (Figure 2A). From the similarity plot, six recombination breakpoints were identified; they were located in NSP2 to NSP3 (3759–5209 nt), NSP9 (8460–9340 nt), and GP3 to N (12,906–14,992 nt). 

Then, the recombination events of the 88 PRRSV-1 full-length genomes from GenBank were identified. First, all strains were preliminarily screened for recombination using RDP software, and recombination events were retained with three recombination signals in seven methods (Table S3). In order to exclude possible false positive results detected by RDP software, SimPlot software was used to perform a second recombination screening and recombination breakpoint location. Specifically, when the similarity between the minor parent and the recombinant was higher than that of the major parent, and the similarity was above 90%, it was considered as a recombination event. (Table S4). The final recombination events (recombination parental strains and breakpoints) were determined after taking into consideration both the RDP and SimPlot results. Of the 91 full-length sequences including three clinical samples, 24 strains were recombined (26%) and all recombinants belonged to subtype 1. The locations of recombination breakpoints were determined according to the positions of PRRS-FR-2005-29-24-1, which was the longest strain of the 91 complete genomes (Figure 2B). The high-frequency recombination regions of the identified PRRSV recombinants were distributed in NSP2 and GP2 to GP4, for which the recombination frequency was greater than or equal to 25%, and five recombination events involving vaccine strains.

### 3.5. Comparison of NSP2 at the Amino Acid Level

**Figure 3:**
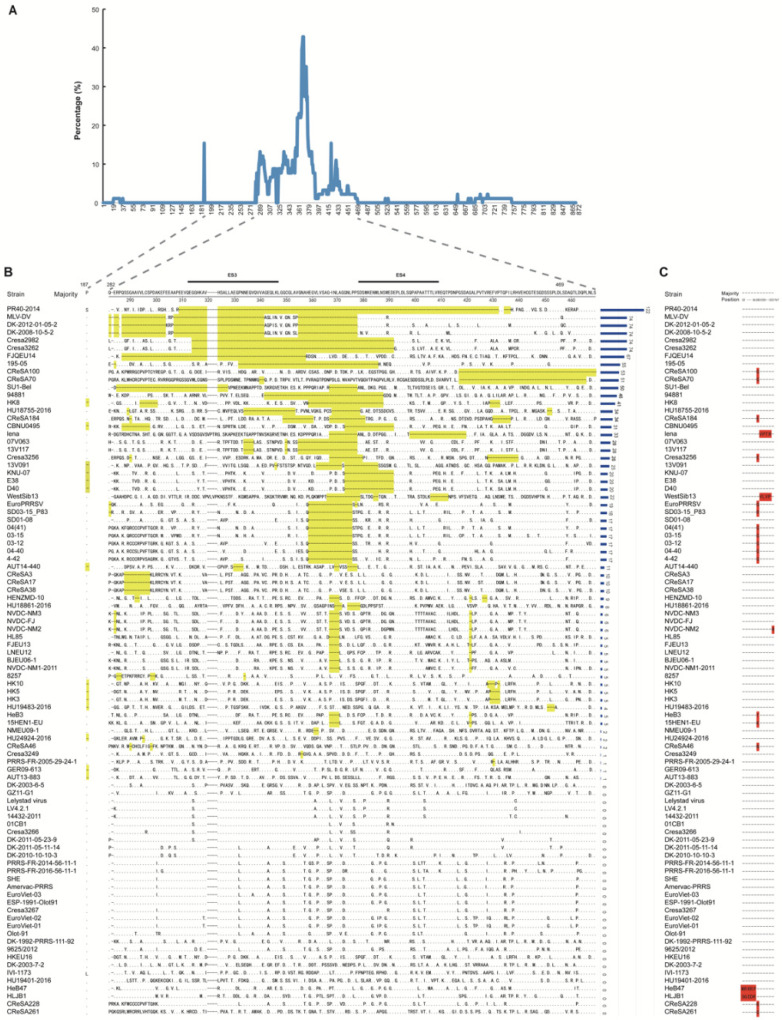
Comparison of NSP2 at the amino acid level: (**A**) The percentage of amino acid deletions at each position in the NSP2 region (aa positions 1–872). (**B**) Distribution of the major amino acid deletion regions of NSP2 (aa positions 187 and 282–469). The deleted amino acids are shaded in yellow. The corresponding number of deletions for each strain in major amino acid deletion regions is shown on the right. Underlined regions show B-cell epitope sites (ES) identified in previous studies [30]. (**C**) Distribution of the amino acid insertion positions of NSP2. The insertion amino acids are shaded in red.

To systematically analyze the NSP2 deletion characteristics of PRRSV-1, NSP2 sequences were intercepted from 91 complete genome sequences. The deletion rate of each amino acid site was calculated (Figure 3A) and the main deletion region was shown in Figure 3B. The deletions number of amino acids in NSP2 for each strain was shown in Table S5. Group 1 (EuroPRRSV, SD03-15_P83, SD01-08, 03-12, 03-15, 04-40, 04-41, and 4-42) had a 17-aa consistent deletion region (at aa position 359 to 375), which is a well-known marker deletion in North American PPRSV-1 strains [16]. Group 2 (MLV-DV, DK-2008-10-5-2, and DK-2012-01-05-2) had similar deletion patterns at aa position 282, 284 to 285, 287 to 303, 307 to 319, 324 to 341, and 355 to 377. Group 3 (NVDC-NM3, NVDC-FJ, NVDC-NM2, HL85, FJEU13, LNEU12, BJEU06-1, and NVDC-NM1-2011) had similar deletion patterns at aa position 367 to 370 and 421. The most variable region of NSP2 in PRRSV-1 samples was located between epitope site 3 (ES3, at aa position 312 to 347) and epitope site 4 (ES4, at aa position 378 to 409).

The deletion numbers of the NSP2 for each strain at aa position 187 and 282 to 469 were calculated. The strains with more deletions in the NSP2 region were PR40-2014 (*n* = 122), MLV-DV (*n* = 74), DK-2012-01-05-2 (*n* = 74), DK-2008-10-5-2 (*n* = 74), Cresa2982 (*n* = 74), and Cresa3262 (*n* = 74). Strains with deletion numbers greater than 11 amino acids were isolated in the United States, Spain, and South Korea. Additionally, only a few strains had discontinuous amino acid insertions at position 32 to 36, 283, 320 to 323, and 767 (Figure 3C). However, there was no obvious distribution characteristic.

### 3.6. Phylogenetic Analysis of Three Samples Based on the Complete Genome Sequences 

**Figure 4:**
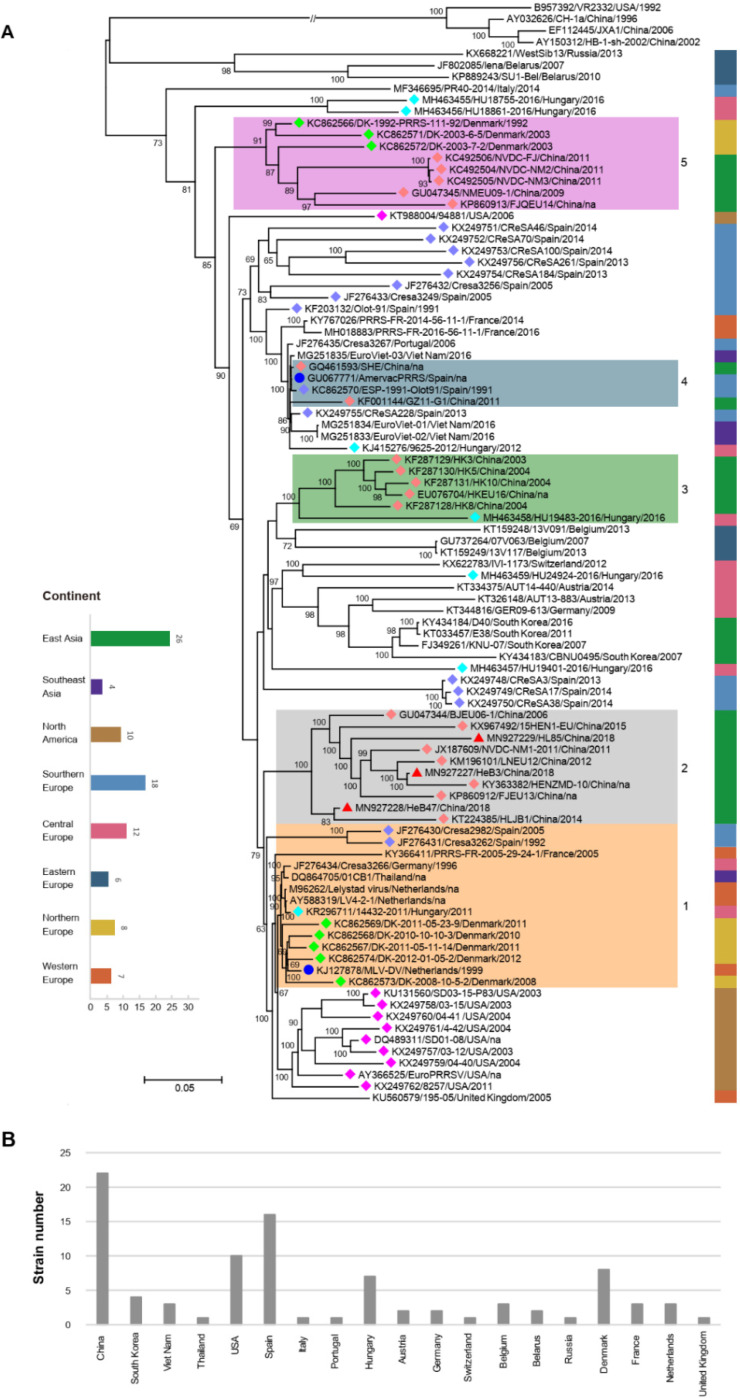
Phylogenetic analysis of three samples based on complete genome sequences: (**A**) Phylogenetic tree based on complete genome sequences of PRRSVs. Information concerning each isolate includes the accession number, name, country, and year. The samples from this study are indicated by red triangles (▲). The vaccine strains are marked by blue dots (●). Strains from different countries are marked with diamonds of different colors (five different colored diamonds). The colors on the branches represent the different introductions. The continent of the strain isolation is shown by the different colored bars, as indicated in the legend. The total number of strains per continent is shown on the left. (**B**) The number of strains researched in each country.

To explore the evolutionary process of the PRRSV-1, RAxML-NG was used to create a phylogenetic tree. The phylogenetic tree is presented in Figure 4, where strains from the same country, marked with the same color diamond (number more than 4), tended to cluster on the same branch (Figure 4A). The continent of each strain was labeled via the corresponding color bar. The strains from North America were clustered together (except KT988004/94881/USA/2006), while strains from other continents did not show significant clustering. LV-like strains were the most predominant and were prevalent in many continents. 

Strains in the same cluster but from different countries indicated that there may have been four introduction events in China. Before 2006, strains represented by LV-like strains (shown in the orange box numbered 1) were introduced into China, became widespread, and evolved independently (shown in the gray box numbered 2). Secondly, PRRSV strains that are similar to that in Hungary were introduced into China (shown in the green box numbered 3). Thirdly, a small-scale introduction of PRRSV strains that are similar to Amervac PRRS vaccine strains can occur in China (shown in the blue box numbered 4), which was reported previously [31]. The fourth introduction was prior to 2009, when PRRSV strains that are similar to that in Denmark were introduced into China, which may be related to swine introduction in China from Denmark (shown in the purple box numbered 5). 

Of all PRRSV-1 complete genomes, East Asia, Southern Europe, and Central Europe had the largest numbers of strains, with 26, 18, and 12, respectively (Figure 4A). In addition, the number of strains in China, the United States, and Spain occupied the top three (Figure 4B). 

### 3.7. Phylogenetic Analysis of Three Samples Based on the ORF5 Genome Sequences 

**Figure 5:**
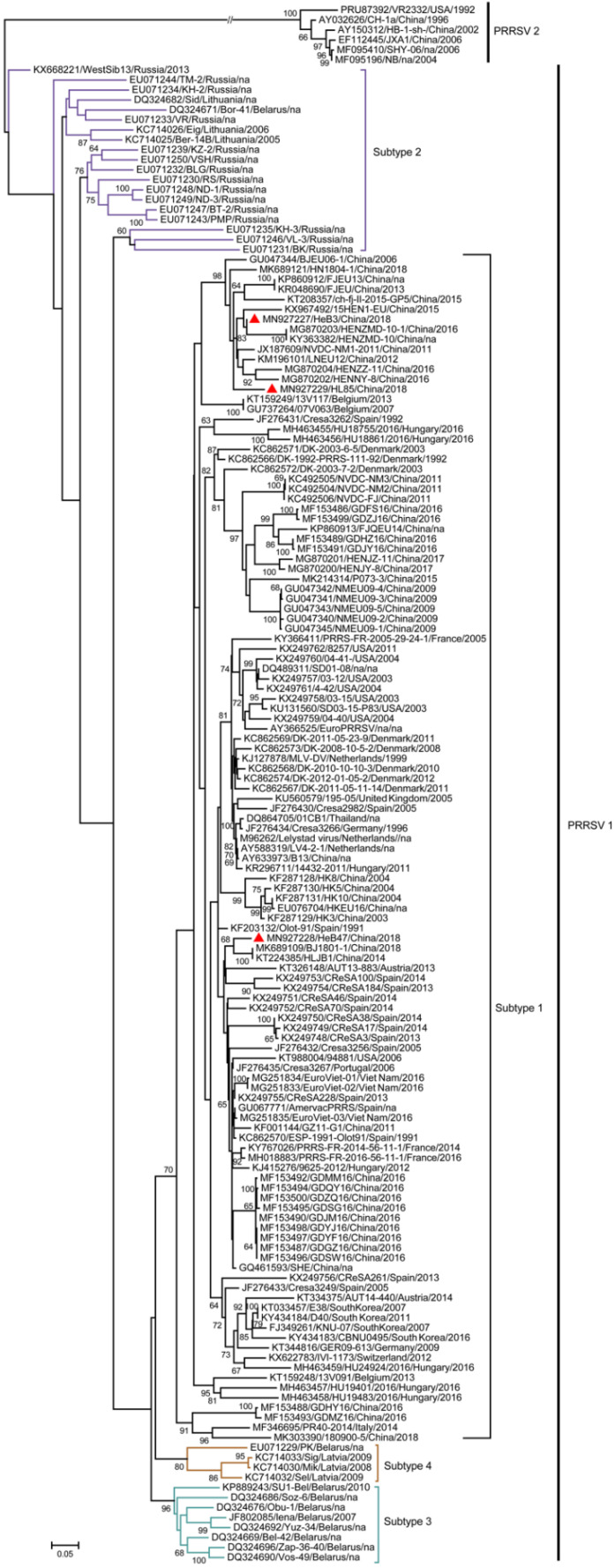
Phylogenetic analysis of three samples based on ORF5 sequences: The samples from this study are indicated by red triangles (▲). Branches highlighting the subtypes of PRRSV-1 strains are marked with different colors.

Based on the phylogenetic analysis of 156 ORF5 nucleotide sequences, 150 PRRSV-1 strains fell into four lineages including subtypes 1 (*n* = 119), 2 (*n* = 19), 3 (*n* = 8), and 4 (*n* = 4) (Figure 5). Strains HL85, HeB3, and HeB47, as well as all Chinese PRRSV-1 strains (*n* = 53), belonged to subtype 1. Interestingly, EuroPRRSV, SD03-15_P83, SD01-08, 03-12, 03-15, 04-40, 04-41, and 4-42, which had similar deletion patterns, were in the same cluster.

### 3.8. Establishment of RT-qPCR for PRRSV-1 Samples

**Figure 6:**
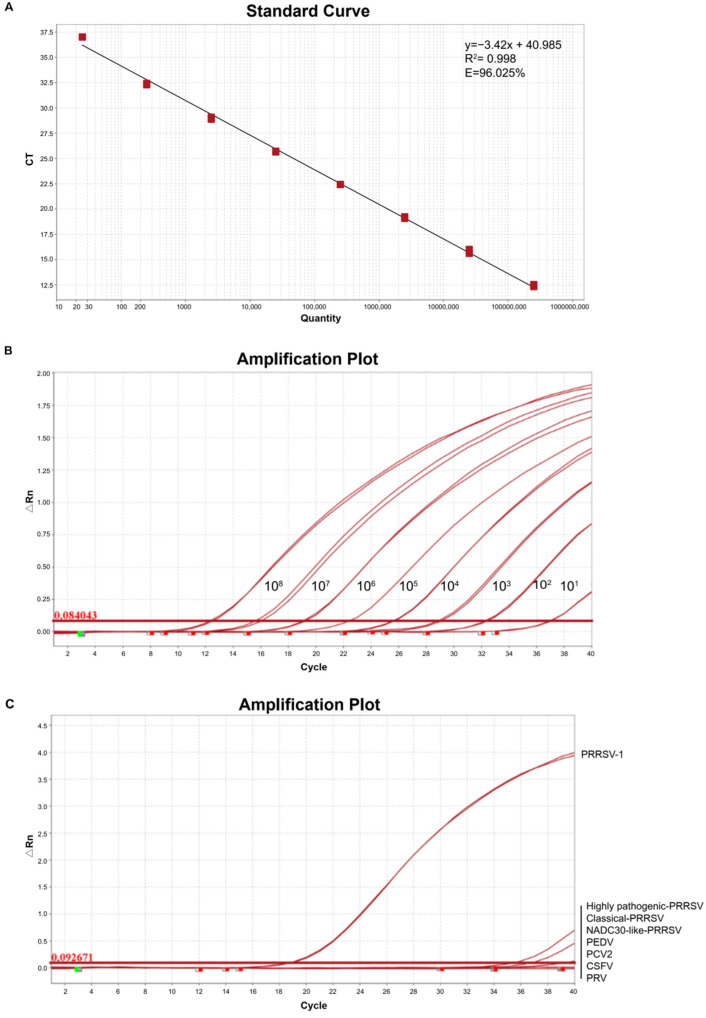
Establishment of RT-qPCR for PRRSV-1 samples: (**A**) Standard curve generated from RT-qPCR assay of the dilution series of standard plasmids. Each data point represents triplicate values and the error bars represent the standard deviation. Linear equation is y = −3.42x + 40.99, “y” represents the threshold cycle, and “x” represents the natural log of concentration. (**B**) Sensitivity analysis of RT-qPCR assay. Standard plasmids of different dilutions (2.5 × 10^1^–2.5 × 10^8^ copies/μL) were used to evaluate the sensitivity. The RT-qPCR has a detection limit of 25 copies/μL. (**C**) Sensitivity analysis of RT-qPCR assay. PRRSV-1, PRRSV-2 (including highly pathogenic-PRRSV, classical-PRRSV, and NADC30-like-PRRSV), PEDV, PCV2, CSFV, and PRV were used as templets.

In order to strengthen monitoring of PRRSV-1 during the importation process and in pig herds, a general and more sensitive method RT-qPCR method for PRRSV-1 was proposed. The standard curve was conducted by standard plasmids of different dilutions (2.5 × 10^1^–2.5 × 10^8^ copies/μL) per reaction. As shown in Figure 6A, the standard curve had an amplification efficiency (Eff %) of 96.03%, a correlation coefficient R^2^ of 0.99, a slope of −3.42, and an intercept of 40.99. For sensitivity analysis, the detection limit of the RT-qPCR was detected by 10-fold serial dilution of standard plasmids. As shown in Figure 6B, the detection limit of the RT-qPCR was 25 copies/μL. For the specificity analysis, nucleic acids extracted from PRRSV-1, PRRSV-2, PEDV, PCV2, CSFV, and PRV were tested by the RT-qPCR assay. As shown in Figure 6C, only PRRSV-1 tested positive with no cross-reactions with other swine pathogens. The results indicated that the RT-qPCR assay had good specificity for PRRSV-1. To evaluate the inter-assay reproducibility and the intra-assay repeatability of RT-qPCR assay, high, medium, and a low dose of standard plasmids (2.5 × 10^6^, 2.5 × 10^4^, and 2.5 × 10^2^ copies/μL) were detected by the RT-qPCR assay. As shown in Table 2, the inter-assay CV ranged from 0.12 to 0.63% and the intra-assay CV ranged from 0.23 to 0.49%. The inter-assay CV and intra-assay CV were both less than 1%, indicating that the developed RT-qPCR assay had good repeatability and reproducibility.

## 4. Discussion

In our clinical sample monitoring of PRRSV-1, three positive samples were detected, which are able to be successfully isolated on cells due to the low virus content or RNA degradation caused by long-term storage of tissue samples. Subsequently, we completed the whole genome sequencing of the three samples for subsequent data analysis. In this study, during 1991–2018, 91 PRRSV-1 complete genomes and 150 PRRSV-1 ORF5 sequences were analyzed to determine their genetic variations, recombination, NSP2 polymorphisms, and evolutionary dynamics. In the analysis of the structural proteins, it was found that GP3 and GP4 were relatively poorly conserved (Table 1), containing a hypervariable region in the overlapping region of GP3 and GP4. In detail, HL85 had a unique deletion of 11 amino acids in the overlapping region of GP3 and GP4, while the other structural proteins were relatively conserved. In previous studies, GP3 and GP4 proteins have different degrees of deletion or termination. It was also reported that there were mutational hotspots at aa position 237 to 252 and 57 to 72 of GP3 and GP4, respectively, in the overlapping region of GP3 and GP4 [32].

During the recombination analysis, we checked the recombination breakpoints in the PRRSVs sequenced in this study. The results showed that these were not adjacent to the corresponding primer-binding sites, which suggested that all recombinant strains comprised field recombination events rather than laboratory artifacts. In addition, RDP and SimPlot were adopted for recombination detection, to exclude the sequences that did not meet the two above conditions. Details of the recombination test that meets both software requirements were shown in Tables S3 and S4. Notably, the high-frequency recombination regions were concentrated in NSP2 and GP2 to GP4. Nsp2 is the most variable protein, and its amino acid deletion is very common, but it does not affect the pathogenicity of PRRSV [33]. GP2 and GP4 are the major determinants of arterivirus entry into cultured cells [34]. Recombination of PRRSV in these regions may be associated with increased cellular tropism, which presumably makes it easier to survive and spread, and ultimately drives the pathogenesis of PRRSV. The atomic force microscopy (AFM) technology may help us understand the attachment of viral proteins to cellular receptors [35], which needs to be further explored.

Extensive recombination of PRRSV may reduce the protective effect of vaccines and also bring difficulties to epidemiological surveillance. Recombination between PRRSV field strains has been extensively reported for PRRSV-2 [36,37] and less frequently for PRRSV-1 [38,39]. Recombination events associated with the vaccine strain have occurred in France, China, and Denmark [18,19,40]. In our recombination analysis, five recombination events involving vaccine strains were detected, including one case in China, which may be related to the use of vaccines in recent years. In previous studies, PRRSV-1 strains recombination has been found to occur not only between vaccine strains and wild strains, but even between two vaccine strains. Specifically, a double recombination event between a diverse field strain of PRRSV and a vaccine or ‘‘vaccine-like’’ strain was identified [17]. Additionally, mosaic strains combining strain VP-046BIS (major parent) and strain DV (minor parent) at nucleotide position 500 to 1370, 3646 to 4272, and 4972 to 8430, all located in ORF1, which encodes the viral RNA replicase [19]. Moreover, a field recombinant strain derived from two MLVs demonstrated high viremia, shedding, and transmission capacities [41,42]. Since the safety of vaccines in the field is a particular concern, viral inspection before mixing herds of swine should be taken to avoid the recombination of PRRSV vaccine strains under field conditions and to use MLVs more carefully.

In the 91 NSP2 sequences, there were different deletion numbers ranging from 0 to 148 amino acids. In previous studies, deletions of up to 74 amino acids in the NSP2 variable region have been described for some other PRRSV-1 strains [39]. In a recent study, six B-cell epitope sites (ES) have been identified in the NSP2 of PRRSV-1. Strikingly, we found that the high-frequency deletion regions of NSP2 were concentrated between ES3 and ES4, which may change the conformation of the epitopes. Some studies have found that the immunodominant epitopes of NSP2 play an important role in regulating the porcine humoral immune response, and the high-frequency deletion regions of NSP2 may affect the pathogenic mechanisms [43,44]. Eight strains isolated from the United States had a continuous “17-aa deletion”, which is a well-known marker deletion in North American PPRSV-1 viruses [16]. Remarkably, they gathered in the same cluster in both complete genome-based and ORF5-based phylogenetic trees, which may have descended from a common evolutionary ancestor. Amino acid deletions in the NSP2 region are common, but are not a determinant of the pathogenicity of PRRSVs [45]. Mutations in a specific part of NSP2 could alter the cellular and/or tissue tropism of PRRSVs. HB-2(sh)/2002, with amino acid deletions at aa position 470 to 481 of NSP2, only affected the primary PAM cells involved in reproduction and did not affect the Marc-145 cells [46]. However, almost all highly-pathogenic PRRSVs (HP-PRRSVs), which have discontinuous amino acid deletions at aa position 481 and 532 to 560 of NSP2, quickly differentiate into Marc-145 cells [2,47].

Although it is mainly prevalent in European countries [13,48], PRRSV-1 has been introduced in non-European countries in recent years, including the USA, Canada, South Korea, and China [16,49,50]. The introduction of different mutated strains can increase genetic diversity and lead to a series of disease outbreaks [51,52]. The phylogenetic analyses based on complete genomes showed that the distribution of the strains had a certain geographical tendency, and those in the same country tended to be clustered together. This is consistent with the previous study [26]. Strains from different countries were distributed in one cluster, and we speculated that there have been four cross-border transmission events, which may be related to the introduction of swine in China from other countries. With the continuous evolution and enrichment of strains, LV-like strains occupy the largest number and are prevalent across multiple continents. Since there was no background control, we were unsure whether a real predominance of LV-like strains or data influenced by selection bias in strain characterization produced this observation. From the ORF5-based phylogenetic tree, it can be found that the subtyping of the virus is related to the countries. For example, only subtype 1 has spread outside Europe, while the remaining subtypes are limited to Eastern European countries, which may be related to the route of introducing pigs and pig breeding scales. Although PRRSV-1 has not yet had a significant impact on the pig industry in China, its high variability and recombination make it necessary to strengthen surveillance to prevent the spread of PRRSV-1. Subsequently, we established a general and more sensitive RT-qPCR method to provide an accurate and fast program for the importation process and PRRSV-1 monitoring in pig herds. In addition, the established optical genome mapping (OGM) technology provides a good platform for unambiguously identifying structural variants of genome sequences.

## 5. Conclusions

In summary, three PRRSV-1 strains in China were full-length sequenced in the present study, one of which was suspected to be a recombinant, and another had an 11-aa continuous deletion in the overlapping region of GP3 and GP4. Furthermore, all available complete genome sequences from GenBank during 1991–2018 were analyzed. The results revealed high-frequency recombination regions were concentrated in NSP2 and GP2 to GP4 and extensive deletions in NSP2. Studying the rules of PRRSV recombination and exploring the impact of vaccine strains on PRRSV recombination can provide new ideas for next-generation vaccines of recombination insensitive. Using the characteristics of NSP2 deletion and designing specific primers or probes for important signature regions of NSP2, it is helpful to develop rapid diagnostic reagents to achieve rapid detection and genotyping. The present work contains valuable information concerning PRRSV-1 and suggests that it is necessary to strengthen the surveillance of PRRSV-1.

## Figures and Tables

**Table 1:** Percentages of nucleotide and amino acid identity of three samples compared with Lelystad virus strain.

	% of Identity to Lelystad Virus		
	HeB3	HeB47	HL85
Complete genome (nt)	91.3	93.8	88.4
pp1a (aa)	89.8	92.3	78.7
pp1ab (aa)	97.5	98.4	96.6
GP2 (aa)	94	94.8	90.4
GP3 (aa)	90.6	92.9	78.9
GP4 (aa)	91.2	90.7	89.5
GP5 (aa)	89.6	91.6	87.6
M (aa)	96.5	98.3	96.5
N (aa)	95.3	96.1	91.5

**Table 2:** Inter- and intra- assay reproducibility of RT-qPCR.

Standard Plasmid (Copies/μL)	Reproducibility (Inter-Assay)		Repeatability (Intra-Assay)	
	Mean ± SD	CV	Mean ± SD	CV
High (2.5 × 10^6^)	19.01 ± 0.03	0.16	19.15 ± 0.11	0.49
Medium (2.5 × 10^4^)	25.59 ± 0.16	0.63	25.67 ± 0.06	0.23
Low (2.5 × 10^2^)	32.51 ± 0.04	0.12	32.35 ± 0.09	0.28

## Data Availability

All available data are presented in the article.

## Supplementary Materials

The following supporting information can be downloaded at: https://www.mdpi.com/article/10.3390/vetsci9090450/s1, Figure S1: The amino acid sequences alignment of GP2, M and N structural proteins; Table S1. Information of the whole-genome PRRSV-1; Table S2. Information of the ORF5 of PRRSV-1; Table S3. Summary of possible recombination events of PRRSV-1 identified by RDP; Table S4. Summary of possible recombination events of PRRSV-1 identified by SimPlot; Table S5. Deletion number of amino acid in NSP2.
