# Supplementary material for: Genomic Analysis of Porcine Reproductive and Respiratory Syndrome Virus 1 Revealed Extensive Recombination and Potential Introduction Events in China
Source: Vet Sci. 2022 Aug 23;9(9):450. doi: 10.3390/vetsci9090450 (PMC9505194; doi:10.3390/vetsci9090450)
Supplement: Supplementary file 1 [file vetsci-09-00450-s001.zip › vetsci-1841180-supplementary.pdf]

Figure S1

A. Alignment of GP2

```
Majority       MQWGHCGVKSVSCSWTLSPSSLLVWLILSFSLPYCLGSPSQAGYWSFFSEWFAPRFSVRALPFTLPNYRRSYEGLLPNCRPDVPQFAIKHPLGMFWHMRV
               ---------+---------+---------+---------+---------+---------+---------+---------+---------+---------+
                       10        20        30        40        50        60        70        80        90       100
               ---------+---------+---------+---------+---------+---------+---------+---------+---------+---------+
EuroPRRSV      ........R.A.....P.L...........F..........D.............................................V............
Lelystad virus ..........A.....P.L........P.............D.............................................V............
HL85           .................L....S..S...........LQ............L................................................
HeB3           .................L....................Q.............................................................
HeB47          ....................................................................................................

Majority       SHLIDEMVSRRIYQTMEHSGQAAWKQVVSEATLTKLSRLDVVTHFQHLAAVEADSCRFLSSRLVMLKNLAVGNVSLQYNTTLDRVELIFPTPGTRPKLTD
               ---------+---------+---------+---------+---------+---------+---------+---------+---------+---------+
                      110       120       130       140       150       160       170       180       190       200
               ---------+---------+---------+---------+---------+---------+---------+---------+---------+---------+
EuroPRRSV      .........................H..G........G..I.........................................N.................
Lelystad virus ............................G........G..I...........................................................
HL85           ..................T........I.D.................................................V....................
HeB3           ....................................................................................................
HeB47          ....................................................................................................

Majority       FRQWLISVHASIFSSVASSVTLFIVLWLRIPVLRYVFGFHWPTATRRSS
               ---------+---------+---------+---------+---------
                      210       220       230       240
               ---------+---------+---------+---------+---------
EuroPRRSV      .......................T.......A.............HH..
Lelystad virus ...............................A.............HH..
HL85           .......................V....................I....
HeB3           ..........................................M......
HeB47          ..........................................M......
```

B. Alignment of M

```
Majority       MGSLDDFCNDPTAAQKLVLAFSITYTPIMIYALKVSRGRLLGLLHILIFLNCSFTFGYMTYVHFQSTNRVALTLGAVVALLWGVYSFTESWKFITSRCRL
               ---------+---------+---------+---------+---------+---------+---------+---------+---------+---------+
                       10        20        30        40        50        60        70        80        90       100
               ---------+---------+---------+---------+---------+---------+---------+---------+---------+---------+
EuroPRRSV      ..G...............................................................A...................L.............
Lelystad virus ..G........I........................................................................................
HL85           .................................................F....................................L......V......
HeB47          ....................................................................................................
HeB3           .................................................F...........................................V......

Majority       CCLGRRYILAPAHHVESAAGLHSISASGNRAYAVRKPGLTSVNGTLVPGLRSLVLGGKRAVKRGVVNLVKYGRY
               ---------+---------+---------+---------+---------+---------+---------+----
                      110       120       130       140       150       160       170
               ---------+---------+---------+---------+---------+---------+---------+----
EuroPRRSV      ..........................................................................
Lelystad virus ..........................................................................
HL85           ........................P.................................................
HeB47          ........................................................S.................
HeB3           ........................P.......................................M.........
```

C. Alignment of N

```
Majority       MAGKNQSQKKKKSTAPMGNGQPVNQLCQLLGAMIKSQRQQPRGGQAKKKKPEKPHFPLAAEDDIRHHLTQTERSLCLQSIQTAFNQGAGTASLSSSGKVS
               ---------+---------+---------+---------+---------+---------+---------+---------+---------+---------+
                       10        20        30        40        50        60        70        80        90       100
               ---------+---------+---------+---------+---------+---------+---------+---------+---------+---------+
EuroPRRSV      ....................................................................................................
Lelystad virus ....................................................................................................
HL85           .............A..............M...IM....R........R......................I.............................
HeB3           ............................M.........R........R....................................................
HeB47          ............N....................M..................................................................

Majority       FQVEFMLPVAHTVRLIRVTSTPASQGAN
               ---------+---------+--------
                      110       120
               ---------+---------+--------
EuroPRRSV      .........G...........S.....S
Lelystad virus .....................S.....S
HL85           ......................VG....
HeB3           ..........................V.
HeB47          ..........................V.
```

Figure S1. The amino acid sequences alignment of GP2, M and N structural proteins. (**A**) Alignment of the GP2 region of type 1 PRRSVs. (**B**) Alignment of the M region of type 1 PRRSVs. (**C**) Alignment of the N region of type 1 PRRSVs.

Supplementary Table S1. Information of the whole-genome PRRSV-1.

	Accession number	Strain name	Year	Country
1	KT326148	AUT13-883	2013	Austria
2	KT334375	AUT14-440	2014	Austria
3	JF802085	lena	2007	Belarus
4	KP889243	SU1-Bel	2010	Belarus
5	GU737264	07V063	2007	Belgium
6	KT159248	13V091	2013	Belgium
7	KT159249	13V117	2013	Belgium
8	KX967492	15HEN1-EU	2015	China
9	GU047344	BJEU06-1	2006	China
10	KP860912	FJEU13	na	China
11	KP860913	FJQEU14	na	China
12	KF001144	GZ11-G1	2011	China
13	MN927227	HeB3	2018	China
14	MN927228	HeB47	2018	China
15	KY363382	HENZMD-10	na	China
16	KF287131	HK10	2004	China
17	KF287129	HK3	2003	China
18	KF287130	HK5	2004	China
19	KF287128	HK8	2004	China
20	EU076704	HKEU16	na	China
21	MN927229	HL85	2018	China
22	KT224385	HLJB1	2014	China
23	KM196101	LNEU12	2012	China
24	GU047345	NMEU09-1	2009	China
25	KC492506	NVDC-FJ	2011	China
26	JX187609	NVDC-NM1-2011	2011	China
27	KC492504	NVDC-NM2	2011	China
28	KC492505	NVDC-NM3	2011	China
29	GQ461593	SHE	na	China
30	KC862566	DK-1992-PRRS-111-92	1992	Denmark
31	KC862571	DK-2003-6-5	2003	Denmark
32	KC862572	DK-2003-7-2	2003	Denmark
33	KC862573	DK-2008-10-5-2	2008	Denmark
34	KC862568	DK-2010-10-10-3	2010	Denmark
35	KC862567	DK-2011-05-11-14	2011	Denmark
36	KC862569	DK-2011-05-23-9	2011	Denmark
37	KC862574	DK-2012-01-05-2	2012	Denmark
38	KY366411	PRRS-FR-2005-29-24-1	2005	France
39	KY767026	PRRS-FR-2014-56-11-1	2014	France
40	MH018883	PRRS-FR-2016-56-11-1	2016	France
41	JF276434	Cresa3266	1996	Germany
42	KT344816	GER09-613	2009	Germany

43	KR296711	14432-2011	2011	Hungary
44	KJ415276	9625-2012	2012	Hungary
45	MH463455	HU18755	2016	Hungary
46	MH463456	HU18861	2016	Hungary
47	MH463457	HU19401	2016	Hungary
48	MH463458	HU19483	2016	Hungary
49	MH463459	HU24924	2016	Hungary
50	MF346695	PR40-2014	2014	Italy
51	M96262	Lelystad-virus	na	Netherlands
52	AY588319	LV4.2.1	na	Netherlands
53	KJ127878	MLV-DV	1999	Netherlands
54	JF276435	Cresa3267	2006	Portugal
55	KX668221	WestSib13	2013	Russia
56	KY434183	CBNU0495	2016	South Korea
57	KY434184	D40	2011	South Korea
58	KT033457	E38	2007	South Korea
59	FJ349261	KNU-07	2007	South Korea
60	GU067771	AmervacPRRS	na	Spain
61	KX249753	CReSA100	2014	Spain
62	KX249749	CReSA17	2014	Spain
63	KX249754	CReSA184	2013	Spain
64	KX249755	CReSA228	2013	Spain
65	KX249756	CReSA261	2013	Spain
66	JF276430	Cresa2982	2005	Spain
67	KX249748	CReSA3	2013	Spain
68	JF276433	Cresa3249	2005	Spain
69	JF276432	Cresa3256	2005	Spain
70	JF276431	Cresa3262	1992	Spain
71	KX249750	CReSA38	2014	Spain
72	KX249751	CReSA46	2014	Spain
73	KX249752	CReSA70	2014	Spain
74	KC862570	ESP-1991-Olot91	1991	Spain
75	KF203132	Olot-91	1991	Spain
76	KX622783	IVI-1173	2012	Switzerland
77	DQ864705	01CB1	na	Thailand
78	KU560579	195-05	2005	United Kingdom
79	KX249762	8257	2011	USA
80	KT988004	94881	2006	USA
81	KX249757	03-12	2003	USA
82	KX249758	03-15	2003	USA
83	KX249760	04(41)	2004	USA
84	KX249759	04-40	2004	USA
85	KX249761	4-42	2004	USA
86	AY366525	EuroPRRSV	na	USA

87	DQ489311	SD01-08	na	USA
88	KU131560	SD03-15-P83	2003	USA
89	MG251834	EuroViet-01	2016	Viet Nam
90	MG251833	EuroViet-02	2016	Viet Nam
91	MG251835	EuroViet-03	2016	Viet Nam

Supplementary Table S2. Information of the ORF5 of PRRSV-1.

	Accession number	Strain name	Year	Country
1	GU047340	NMEU09-2	2009	China
2	GU047341	NMEU09-3	2009	China
3	GU047342	NMEU09-4	2009	China
4	GU047343	NMEU09-5	2009	China
5	KR048690	FJEU	2013	China
6	KT208357	ch-fj-II-2015-GP5	2015	China
7	MK214314	P073-3	2015	China
8	MF153486	GDFS16	2016	China
9	MF153487	GDGZ16	2016	China
10	MF153488	GDHY16	2016	China
11	MF153489	GDHZ16	2016	China
12	MF153490	GDJM16	2016	China
13	MF153491	GDJY16	2016	China
14	MF153492	GDMM16	2016	China
15	MF153493	GDMZ16	2016	China
16	MF153494	GDQY16	2016	China
17	MF153495	GDSG16	2016	China
18	MF153496	GDSW16	2016	China
19	MF153497	GDYF16	2016	China
20	MF153498	GDYJ16	2016	China
21	MF153499	GDZJ16	2016	China
22	MF153500	GDZQ16	2016	China
23	MG870202	HENNY-8	2016	China
24	MG870203	HENZMD-10-1	2016	China
25	MG870204	HENZZ-11	2016	China
26	MG870200	HENJY-8	2017	China
27	MG870201	HENJZ-11	2017	China
28	MK303390	180900-5	2018	China
29	MK689109	BJ1801-1	2018	China
30	MK689121	HN1804-1	2018	China
31	AY633973	B13	na	China

Supplementary Table S3. Summary of possible recombination events of PRRSV-1 identified by RDP.

Event	Recombinant	Parental sequence	Breakpoint region		P-Value for the seven detection methods in RDP4						
number	Sequence(s)	Major/Minor	Begin	End	RDP	GENECONV	Bootscan	Maxchi	Chimaera	SiSscan	3Seq
1	HKEU16	HK5/HK10	198*	2091	4.60E-19	2.71E-17	NS	2.43E-07	1.86E-02	5.53E-18	1.26E-11
2	HeB47	BJEU06-1/CReSA228	12500	14000	NS	NS	NS	2.16E-05	8.39E-06	1.05E-17	6.65E-05
3	NVDC-NM1-2011	HeB3/BJEU06-1	588	2541	6.93E-81	3.62E-80	7.68E-92	1.24E-25	2.83E-26	4.44E-34	1.26E-11
3	NVDC-NM1-2011	HeB3/BJEU06-1	6985	9139	3.97E-69	1.22E-69	1.96E-72	5.93E-20	3.97E-19	3.01E-25	2.52E-11
4	HLJB1	Amervac PRRS/BJEU06-1	10126	12766	1.01E-14	NS	NS	6.82E-12	4.55E-18	1.29E-02	3.38E-03
5	DK-2011-05-11-14	LV4.2.1/DK-2010-10-10-3	3110	6590	1.57E-03	NS	1.04E-02	NS	NS	NS	4.96E-02
6	04(41)	SD01-08/SD03-15_P83	518	4420	6.04E-120	7.59E-119	1.19E-99	8.60E-39	2.37E-38	2.42E-49	1.26E-11
7	AUT14-440	E38/DK-2011-05-11-14	1879*	6005	NS	NS	NS	6.43E-09	1.06E-10	1.09E-08	1.26E-11
8	CReSA100	CReSA261/CReSA184	13262	15111*	7.27E-17	NS	4.55E-02	8.98E-06	6.30E-11	9.38E-15	2.52E-11
9	IVI-1173	Lelystad virus/D40	12529	14367	NS	NS	NS	1.75E-05	1.31E-05	NS	2.52E-11
10	HU24924/2016	MLV-DV/KNU-07	12529	14367	NS	NS	NS	1.75E-05	1.31E-05	NS	2.52E-11
11	HU18861/2016	HU18755/2016/DK-2011-05-11-14	3721	4109	5.07E-05	NS	NS	5.72E-05	1.63E-02	NS	5.59E-07
12	04-40	SD01-08/8257	10964	12122	1.31E-08	3.68E-06	1.48E-04	6.25E-03	NS	1.60E-08	1.60E-03
13	AUT13-883	GER09-613/D40	6512	7087	NS	NS	NS	7.91E-08	2.28E-09	NS	2.52E-11
14	CReSA70	Amervac PRRS/CReSA261	586	1532	1.85E-29	NS	NS	1.74E-12	1.10E-14	NS	1.13E-10
15	HU19483/2016	Cresa3266/KNU-07	802	1497	3.48E-14	NS	4.20E-04	3.20E-04	2.61E-04	NS	4.35E-09
16	Cresa3262	Cresa2982/Lelystad virus	11764	12421	NS	6.26E-24	NS	2.07E-09	5.72E-03	6.03E-10	2.52E-11
17	Cresa3267	Amervac PRRS/LV4.2.1	6380	6927	4.79E-18	7.38E-15	4.78E-18	1.74E-05	1.20E-04	3.43E-05	2.52E-11
18	MLV-DV	01CB1/CReSA228	11936	12444	2.84E-17	1.59E-14	2.61E-17	1.61E-04	9.20E-05	1.03E-03	7.56E-11
19	BJEU06-1	NVDC-NM1-2011/NVDC-FJ	12957	13467	4.21E-12	NS	1.06E-04	NS	NS	NS	8.93E-09
20	DK-2010-10-10-3	Lelystad virus/DK-2012-01-05-2	13533	14126	NS	NS	NS	6.35E-07	1.83E-06	3.65E-10	NS
21	CBNU0495	KNU-07/8257	12100	12676	1.95E-03	NS	1.19E-02	4.77E-07	8.18E-06	NS	7.56E-09
22	CReSA261	Olot/91/CReSA70	12522	12878	7.62E-15	1.47E-08	4.10E-14	1.15E-06	1.06E-06	5.35E-06	1.96E-08

23	GER09-613	D40/PRRS-FR-2016-56-11-1	5080*	5549	3.65E-04	NS	NS	1.72E-05	8.39E-05	NS	NS
24	DK-2008-10-5-2	MLV-DV/NMEU09-1	3575	3996*	1.68E-07	NS	6.32E-03	3.69E-03	1.68E-02	NS	1.45E-05

Supplementary Table S4. Summary of possible recombination events of PRRSV-1 identified by SimPlot.

	Strain	Major	Minor	Region 1			Region 2			Region 3			Region 4			Region 5			Region 6		
	name	parent	parent	Left	Right	Length	Left	Right	Length	Left	Right	Length	Left	Right	Length	Left	Right	Length	Left	Right	Length
1	HKEU16	HK5	HK10	1	2145	2144	2395	2739	344	7680	8152	472	9949	10546	597	12100	12653	553	13096	13362	266
2	HeB47	BJEU06-1	CReSA228	3759	5209	1450	8460	9340	880	12906	14992	2086									
3	NVDC-NM1-2011	HeB3	BJEU06-1	548	2617	2069	7057	9250	2193												
4	HLJB1	Amervac PRRS	BJEU06-1	6598	7749	1151	10193	12802	2609												
5	DK-2011-05-11-14	LV4.2.1	DK-2010-10-10-3	2199	2550	351	3102	6562	3460												
6	04(41)	SD01-08	SD03-15_P83	518	4420	3902															
7	AUT14-440	E38	DK-2011-05-11-14	3836	6070	2234	8561	8870	309	11494	12605	1111									
8	CReSA100	CReSA261	CReSA184	12242	15111	2869															
9	IVI-1173	Lelystad virus	D40	12529	14367	1838															
10	HU24924/2016	MLV-DV	KNU-07	12917	13520	603															
11	HU18861/2016	HU18755/2016	DK-2011-05-11-14	3744	4145	401															
12	04-40	SD01-08	8257	10962	12378	1416															
13	AUT13-883	GER09-613	D40	5850	6122	272	6431	7110	679	8378	8652	274									
14	CReSA70	Amervac PRRS	CReSA261	591	1571	980	12512	12849	337												
15	HU19483/2016	Cresa3266	KNU-07	802	1497	695															
16	Cresa3262	Cresa2982	Lelystad virus	11764	12421	657															
17	Cresa3267	Amervac PRRS	LV4.2.1	6380	6927	547															
18	MLV-DV	01CB1	CReSA228	11936	12444	508															
19	BJEU06-1	NVDC-NM1-2011	NVDC-FJ	12957	13467	510															
20	DK-2010-10-10-3	Lelystad virus	DK-2012-01-05-2	13559	14113	554															
21	CBNU0495	KNU-07	8257	12300	12768	468															
22	CReSA261	Olot/91	CReSA70	12514	12981	467															

23	GER09-613	D40	PRRS-FR-2016-56-11-1	5080	5549	469
24	DK-2008-10-5-2	MLV-DV	NMEU09-1	3575	3996	421

Supplementary Table S5. Deletion number of amino acid in NSP2.

	Strain	Deletion number (aa)
1	PR40-2014	148
2	CReSA100	106
3	MLV-DV	74
4	DK-2012-01-05-2	74
5	DK-2008-10-5-2	74
6	Cresa2982	74
7	Cresa3262	74
8	FJQEU14	68
9	195-05	55
10	CReSA70	51
11	SU1-Bel	50
12	94881	47
13	HK8	41
14	HU18755-2016	34
15	CReSA184	34
16	CBNU0495	31
17	lena	33
18	07V063	28
19	13V117	28
20	Cresa3256	26
21	13V091	25
22	KNU-07	20
23	E38	20
24	D40	20
25	WestSib13	23
26	EuroPRRSV	19
27	SD03-15-P83	18
28	SD01-08	17
29	04(41)	17
30	03-15	17
31	03-12	17
32	04-40	17
33	4-42	17
34	AUT14-440	13
35	CReSA3	10
36	CReSA17	10
37	CReSA38	10
38	HENZMD-10	9
39	HU18861-2016	8
40	NVDC-NM3	6
41	NVDC-FJ	6
42	HL85	5

43	FJEU13	5
44	LNEU12	5
45	BJEU06-1	5
46	NVDC-NM1-2011	5
47	NVDC-NM2	6
48	8257	5
49	HK10	5
50	HK5	5
51	HK3	5
52	HU19483-2016	5
53	HeB3	5
54	15HEN1-EU	5
55	DK-2003-6-5	3
56	NMEU09-1	2
57	HU24924-2016	2
58	CReSA46	3
59	GZ11-G1	1
60	Cresa3249	1
61	PRRS-FR-2005-29-24-1	1
62	GER09-613	1
63	AUT13-883	1
64	Lelystad-virus	0
65	LV4.2.1	0
66	14432-2011	0
67	01CB1	0
68	Cresa3266	0
69	DK-2011-05-23-9	0
70	DK-2011-05-11-14	0
71	DK-2010-10-10-3	0
72	PRRS-FR-2014-56-11-1	0
73	PRRS-FR-2016-56-11-1	0
74	SHE	0
75	Amervac-PRRS	0
76	EuroViet-03	0
77	ESP-1991-Olot91	0
78	Cresa3267	0
79	EuroViet-02	0
80	EuroViet-01	0
81	Olot-91	0
82	DK-1992-PRRS-111-92	0
83	9625/2012	0
84	HKEU16	0
85	DK-2003-7-2	0
86	IVI-1173	0

87	HU19401-2016	0
88	CReSA228	0
89	CReSA261	0
90	HeB47	0
91	HLJB1	0
